# The stiffness of three-dimensional biomimetic matrices regulates insulin secretion via the Piezo1 mechanosensitive ion channel

**DOI:** 10.3389/fchem.2026.1981529

**Published:** 2026-10-09

**Authors:** Xi Chen, Zhen Wang, Jiahao Gu, Yan Xue, Yang Xu, Yan Huang, Dongzhi Wang

**Affiliations:** 1 Department of Hepatobiliary and Pancreatic Surgery, Affiliated Hospital of Nantong University, Nantong, China; 2 Key Laboratory of Neuroregeneration of Jiangsu and Ministry of Education, Co-innovation Center of Neuroregeneration, Nantong University, Nantong, China; 3 Research Center of Molecular Medicine, Nantong Health Vocational College, Nantong, China

**Keywords:** diabetes, hyaluronic acid methacrylate, insulin secretion, matrix stiffness, Piezo1

## Abstract

**Introduction:**

The extracellular matrix (ECM) within the pancreatic microenvironment plays an integral role in the pathogenesis of diabetes. However, how matrix stiffness regulates islet β-cell function through mechanosensitive ion channels remains poorly understood.

**Methods:**

A three-dimensional biomimetic islet culture system with graded matrix stiffness was established using hyaluronic acid methacrylate (HAMA) hydrogels with different crosslinking times. MIN6 insulin-secreting cells were encapsulated in soft and stiff HAMA matrices, and cell viability, Piezo1 expression, intracellular Ca^2+^ levels, β-cell function-related gene expression, and glucose-stimulated insulin secretion (GSIS) were evaluated. Pharmacological modulation of mechanosensitive ion channels and Piezo1 knockdown were used to investigate the potential role of Piezo1 in stiffness-dependent β-cell responses. RNA sequencing and pathway enrichment analyses were performed to identify signaling pathways associated with matrix stiffness. In addition, stiff or soft matrix-encapsulated MIN6 cells were transplanted into diabetic mice to evaluate in vivo glucose regulation and biocompatibility.

**Results:**

Stiffer HAMA hydrogels significantly increased Piezo1 expression in MIN6 cells and were associated with elevated intracellular Ca^2+^ levels. Compared with the soft matrix, the stiff matrix significantly increased the expression of β-cell function-related genes, including Insulin, Pdx1, Mafa, and Nkx6-1, and enhanced insulin secretion under both basal and glucose-stimulated conditions. Pharmacological activation of Piezo1 in the soft matrix partially mimicked the effects of the stiff matrix, whereas inhibition of mechanosensitive ion channels reduced insulin-associated signals and β-cell function-related gene expression. RNA-seq and pathway enrichment analyses identified HIF-related signaling and metabolic pathways as potential molecular responses to matrix stiffness. However, the present data did not establish a direct mechanistic relationship between HIF signaling and Piezo1. In diabetic mice, transplantation of stiff matrix-encapsulated MIN6 cells was associated with improved blood glucose control during the observation period and showed good in vivo biocompatibility.

**Discussion:**

These findings indicate that matrix stiffness can modulate β-cell function and glucose-responsive insulin secretion within a three-dimensional microenvironment. Overall, this study provides evidence that biomechanical properties of the three-dimensional ECM may influence β-cell function and highlights matrix mechanics as a potential regulatory component of the islet microenvironment.

## 1 Introduction

Diabetes mellitus is a chronic metabolic disorder characterized by insufficient insulin secretion or defects in insulin utilization, with its pathogenesis closely linked to the dynamic remodeling of the pancreatic islet extracellular matrix (ECM). Under normal physiological conditions, the islet ECM, composed of type IV collagen, laminin, and hyaluronic acid (HA), provides essential mechanical support and biochemical signals to β-cells, maintaining their functional homeostasis. Changes in ECM composition and physicochemical properties can disrupt pancreatic function, contributing to a variety of pancreatic diseases (Zhang et al., 2021). For example, in pancreatic ductal adenocarcinoma (PDAC), a hallmark stromal response leads to excessive deposition of collagen and HA, forming dense fibrotic networks that promote tumor cell invasion and chemoresistance (Zanotelli et al., 2021; Deng et al., 2022; Gong et al., 2023; Pan et al., 2023). In early-stage type 1 diabetes mellitus (T1DM), abnormal accumulation of peri-islet HA attracts inflammatory cell infiltration, exacerbating autoimmune responses and ultimately resulting in β-cell loss (Bluestone et al., 2010). Fibrosis, a critical histopathological feature of pancreatic diseases, progressively increases local tissue stiffness, influencing mechanotransduction pathways between cells and ECM (Di et al., 2023). Although increased stiffness in type 2 diabetes has been shown to disrupt insulin secretion through altered mechanosensitive transcriptional regulation, the role of ECM mechanical properties in regulating β-cell function under physiological conditions remains poorly understood (Song and Ergul, 2006). Understanding how mechanical stress dynamically regulates insulin secretion through specific molecular sensors may offer novel insights into biomechanics-based precision therapies.

The mechanical properties of the ECM, including stiffness, viscoelasticity, and plasticity, are essential for tissue homeostasis and cellular behavior (Nia et al., 2020; Cukierman and Bassi, 2010). Stiffness, quantified as the elastic modulus, reflects the ECM’s resistance to deformation and its mechanical support for cells (Tilghman et al., 2010). Physiological peri-islet ECM stiffness (0.1–1.5 kPa, typical of soft tissues) provides an optimal microenvironment for maintaining β-cell metabolic sensitivity. In contrast, pathological conditions such as PDAC or chronic pancreatitis induce abnormal ECM remodeling, significantly increasing stromal stiffness (Pandol et al., 2009). These mechanical changes not only compromise the structural integrity of islets but also impair cellular function via mechanotransduction pathways. β-Cell mechanosensing involves multiple mechanisms, including the integrin-focal adhesion kinase (FAK) signaling axis and mechanosensitive ion channels. Integrins, as transmembrane receptors, bind ECM ligands (e.g., fibronectin, laminin), triggering FAK phosphorylation and activating downstream RhoA/ROCK pathways that regulate cytoskeletal reorganization and gene expression (Samuel et al., 2011; Zhao and Guan, 2011). Concurrently, mechanosensitive ion channels mediate transmembrane ion flux by sensing changes in membrane tension, activating β-cell electrical activity and voltage-gated calcium channels (VGCCs), which in turn promote insulin vesicle exocytosis (Tranter et al., 2023). This mechano-electrical coupling highlights the dynamic interplay between ECM mechanics and β-cell secretory function.

Recent advances have clarified the functional mechanisms of Piezo1, a mechanosensitive nonselective cation channel (Qiu et al., 2023; Coste et al., 2010). Emerging research indicates that Piezo1 activation in CCl4 and CDAHFD-induced hepatic fibrosis models enhances macrophage efferocytosis, reduces collagen deposition and α-smooth muscle actin (α-SMA) expression, and mitigates fibrogenesis. Conversely, macrophage Piezo1 activation promotes M1 polarization and hepatic stellate cell activation, creating a profibrotic microenvironment (Greuter et al., 2022; Leng et al., 2022). In cardiac tissue, Piezo1 regulates myocardial contractility via Ca^2+^-ROS signaling, with moderate activation enhancing compensatory capacity, while hyperactivation induces arrhythmias (Pethő et al., 2019). Piezo1 knockout impairs cardiac pumping and leads to dilated cardiomyopathy. These findings indicate Piezo1’s dual role in mechanotransduction—physiological mechanical stimuli preserve cellular homeostasis, while dysregulated activation contributes to disease progression. Although β-cell Piezo1 hyperactivation has been implicated in diabetic insulin secretion dysfunction, strategies for dynamically modulating Piezo1 through matrix stiffness remain undefined (Jiang et al., 2025; Kim et al., 2022). Additionally, conventional biomechanical studies often rely on 2D culture systems, which fail to mimic the 3D ECM architecture and the spatial transmission of *in vivo* mechanosignals (Habanjar et al., 2021; Fu et al., 2023; Clevers, 2016). Therefore, developing 3D islet-mimetic culture systems with tunable stiffness is essential to exploring mechano-metabolic coupling.

HA, a natural glycosaminoglycan present in mammalian ECM, is renowned for its biocompatibility, low immunogenicity, and angiogenic properties mediated by endothelial receptors (Marinho et al., 2021). This makes HA a promising candidate for islet regenerative engineering. Notably, HA lacks integrin-binding RGD motifs, signaling primarily through mechanosensitive ion channels rather than integrin pathways. Methacrylated HA (HAMA), modified with methacrylate groups, forms 3D hydrogels via 405 nm UV-initiated crosslinking with LAP photoinitiator. HAMA hydrogels retain HA’s biocompatibility and hydrophilicity while enabling precise control over stiffness through crosslinking density modulation (Lu et al., 2025), making them an ideal platform for simulating islet micromechanics and investigating stiffness-dependent β-cell regulation.

Our findings establish a stiffness-responsive three-dimensional HAMA microenvironment that modulates β-cell function through Piezo1-associated mechanosensing and Ca^2+^ signaling. By integrating biomimetic matrix engineering with pharmacological and genetic interrogation, transcriptomic profiling, and *in vivo* validation, this strategy provides a novel framework for linking extracellular matrix mechanics to β-cell secretory function. Such mechanically responsive regulation of the islet microenvironment may offer a promising avenue for enhancing glucose-responsive insulin secretion and developing biomechanical strategies for diabetes therapy.

## 2 Materials and methods

### 2.1 Animal care

All experimental procedures and animal care/handling were conducted in accordance with the guidelines of the Nantong University Institutional Animal Care and Use Committee (approval No. S20220219-007). Healthy 6-week-old male C57BL/6 mice were obtained from the Experimental Animal Center of Nantong University. The mice were housed in a specific pathogen-free (SPF) barrier facility on a 12-hour light/dark cycle, with *ad libitum* access to food and water throughout the experimental period. All animal procedures adhered to the Guidelines for the Care and Use of Laboratory Animals established by Nantong University and were approved by the Animal Ethics Committee of Nantong University.

### 2.2 Hyaluronic acid hydrogel preparation

Lithium phenyl-2,4,6-trimethylbenzoylphosphinate (LAP, 25 mg), a photoinitiator, was dissolved in 10 mL of phosphate-buffered saline (PBS) and incubated at 45 °C for 15 min to prepare a 0.25% (w/v) LAP solution. Then, 100 mg of HAMA (molecular weight: 400 kDa) was added to the solution and dissolved under light-protected conditions at room temperature for 1 h, resulting in a 1% (w/v) HAMA solution. The solution was sterilized via filtration through a 0.22 μm membrane. Hydrogels with biomimetic matrix stiffness gradients were fabricated by adjusting the crosslinking time (10, 15, 30, 45, and 60 s) under 405 nm ultraviolet (UV) light.

### 2.3 Scanning electron microscopy

HAMA hydrogels were frozen at −80 °C for 12 h, followed by lyophilization in a vacuum freeze-dryer for 24 h. The lyophilized samples were mounted on a metal stage, sputter-coated with platinum, and analyzed using field-emission scanning electron microscopy (FE-SEM) at an accelerating voltage of 5 kV. The pore sizes of the hydrogels were quantified using ImageJ software (n = 3).

### 2.4 Mechanical property testing

Cylindrical HAMA hydrogel samples (diameter = 9 mm, height = 10 mm) were placed on a horizontal platform and compressed at a displacement rate of 5 mm/min using an MTS universal testing machine until rupture. Stress-strain curves were plotted, and the apparent Young’s modulus was calculated as the slope of the linear region (5%–15% strain) of the curves.

### 2.5 Cell culture and 3D encapsulation

The mouse pancreatic β-cell line MIN6 was cultured in high-glucose Dulbecco’s Modified Eagle Medium (DMEM) supplemented with 10% fetal bovine serum (FBS, Gibco, Burlington, Ontario, Canada), 100 μg/mL streptomycin, 100 units/mL penicillin, 10 mmol/L HEPES, and 50 μmol/L β-mercaptoethanol. Cells were maintained at 37 °C in a humidified 5% CO_2_ atmosphere. For 3D encapsulation, 1 mL of sterile HAMA solution was mixed with 1 × 10^6^ MIN6 cells to form a homogeneous suspension. The mixture (180 μL/well) was added to a 6-well plate and crosslinked under 405 nm UV light to generate HAMA hydrogels encapsulating MIN6 cells.

### 2.6 Cell viability staining

After 5 days of culture in glass-bottom dishes at predetermined densities, MIN6 cells were washed with PBS to remove residual medium. Calcein-AM and propidium iodide (PI) staining solutions were added, followed by incubation in the dark at 37 °C for 25 min and 5 min, respectively. After PBS washing, cell viability was visualized using a confocal fluorescence microscope (LSM900, Zeiss).

### 2.7 CCK-8 cell proliferation assay

MIN6 cells (2 × 10^3^ cells/well, n = 6) were seeded in 96-well plates. After adhesion, the medium was replaced with 100 μL of hydrogel extracts from different groups and incubated for 24, 48, and 72 h. The extracts were removed, and 10 μL of CCK-8 reagent was added to each well. After 2 h of incubation in the dark, the absorbance at 450 nm was measured using a microplate reader.

### 2.8 RNA extraction and real-time quantitative PCR

Total RNA was extracted from MIN6 cells encapsulated in soft and stiff matrix hydrogels (n = 3) using Trizol reagent, and the RNA concentration was quantified spectrophotometrically. The isolated RNA was reverse transcribed into cDNA using a cDNA synthesis kit (Thermo Fisher Scientific, K1622). Quantitative real-time polymerase chain reaction (qPCR) analysis was performed on the QuantStudio 5 system using Fast SYBR Green qPCR Master Mix, with cDNA as the template. Primers synthesized by Sangon Biotechnology Company are listed in Supplementary Table S1. Gene expression levels were normalized to murine β-actin expression, and comparative quantification was conducted using the 2^−ΔΔCT^ method.

### 2.9 Immunofluorescence (IF)

For IF, MIN6 cells cultured in different matrix hydrogels were fixed with 4% paraformaldehyde at 37 °C for 30 min, followed by PBS washes. Samples were permeabilized with 0.5% Triton X-100 (Sigma-Aldrich, United States) for 15 min, rinsed with PBS, and blocked with 5% bovine serum albumin (BSA) for 2 h. Samples were then incubated with primary antibodies diluted in antibody dilution buffer at 4 °C overnight, followed by three washes with PBS containing 0.1% Tween-20 (PBST). The samples were incubated with corresponding secondary antibodies in the dark at room temperature for 2 h, followed by PBST washes to remove unbound fluorescent conjugates. Nuclei were counterstained with DAPI for 10 min before confocal microscopy imaging. Primary antibodies/reagents used included: Anti-Piezo1 antibody (1:100); Anti-insulin antibody (1:200, Thermo Fisher Scientific, United States); Anti-glucagon antibody (1:200); Alexa Fluor 488-conjugated goat anti-rabbit IgG (1:500, Abcam, United Kingdom); Alexa Fluor 488-conjugated goat anti-mouse IgG (1:500, Abcam, United Kingdom); Alexa Fluor 594-conjugated goat anti-mouse IgG (1:500, Abcam, United Kingdom); DAPI nuclear stain (1:5000, Sigma-Aldrich, D9542). For quantitative fluorescence analysis, images were acquired under consistent imaging conditions within each experimental comparison. Predefined regions of interest (ROIs) were used consistently for fluorescence quantification, and fluorescence intensity was measured using ImageJ. The same image-acquisition settings were applied to the corresponding samples. The measured fluorescence intensity values were used for subsequent statistical analysis.

### 2.10 Glucose-stimulated insulin secretion (GSIS)

For GSIS assays, cell-laden hydrogels from each experimental group were seeded into 6-well plates and cultured in a constant-temperature cell culture incubator for 3 days before starvation treatment. After thorough washing with PBS, the hydrogels were equilibrated in HEPES-buffered Krebs-Ringer bicarbonate solution (KRBH) for 30 min. Subsequently, hydrogel-encapsulated MIN6 cells were incubated in KRBH containing either low glucose (2.5 mM) or stimulatory glucose (25 mM) concentrations for 1 h. Supernatants were then collected for insulin secretion quantification.

### 2.11 Establishment of type 1 diabetic mouse model and subcutaneous transplantation

Streptozotocin (STZ, Sigma-Aldrich, United States) was dissolved in citrate buffer (1:1.32 ratio of citric acid solution to sodium citrate solution) to prepare a 1% (w/v) STZ solution, which was then filtered through a 0.22 μm sterile filter. 6-week-old healthy C57BL/6 mice were fasted for 16 h, weighed, and then administered the freshly prepared STZ solution via intraperitoneal injection at a dose of 150 mg/kg. Diabetes model validation was confirmed if fasting blood glucose levels exceeded 16.7 mmol/L on two consecutive days starting from day 3 post-injection. Six mice per group were depilated in the dorsolateral region, and a 1 cm longitudinal skin incision was made to create a subcutaneous pocket through blunt dissection of loose connective tissue, into which cell-laden hydrogels were implanted.

### 2.12 Dynamic blood glucose monitoring

Starting the day after transplantation, micro whole blood samples were collected from the tail vein of mice, discarding the first drop of blood. Blood glucose was monitored using a glucose meter at a frequency of three times per week. Transplantation was considered successful when non-fasting blood glucose levels remained ≤11.1 mmol/L for three consecutive days.

### 2.13 Intraperitoneal glucose tolerance test (IPGTT)

A 200 mg/mL glucose solution was prepared in saline, and the mice were fasted for 16 h before the experiment, with normal water intake maintained. Fasting blood glucose levels were measured using a handheld glucose meter, with the baseline blood glucose value recorded at 0 min. After stabilizing the mice for a few moments, 2 g/kg body weight of glucose solution was injected intraperitoneally. Blood glucose levels were measured at 15, 30, 60, and 120 min after injection.

### 2.14 Enzyme-linked immunosorbent assay (ELISA) for insulin secretion

Insulin secretion in cell culture supernatants or mouse serum was determined by ELISA using the Mouse Ultrasensitive Insulin Assay Kit (Mercodia AB, 10-1249-01, Sweden), and the results were standardized according to protein content.

### 2.15 Analysis of mRNA sequencing data

mRNA extracted from MIN6 cells encapsulated in soft and rigid matrix hydrogels underwent library preparation and sequencing. RNA integrity was assessed using an Agilent 2100 Bioanalyzer (Agilent Technologies). Barcoded libraries were generated and quantified using the NEBNext Ultra RNA Library Prep Kit (Cat. No. E7530, New England Biolabs). Libraries were pooled based on effective concentrations and sequencing depth requirements, followed by index-coded sample clustering. Paired-end sequencing was then performed on an Illumina platform. Raw sequencing data (raw reads) in FASTQ format were preprocessed using in-house Perl scripts. Clean reads were obtained by removing adapter-containing sequences, poly-N fragments, and low-quality reads. Differential gene expression analysis was conducted using DESeq2 (v1.25.10) in R, retaining genes with non-zero counts in ≥50% of samples. Genes with an adjusted p-value < 0.05 were considered DEGs. Hierarchical clustering of DEGs based on expression profiles was visualized using the DEGreport R package (v1.21.1). Gene Ontology (GO) term enrichment analysis was performed with ClusterProfiler (v3.13.0), with statistical significance determined by a Benjamini-Hochberg-adjusted q-value < 0.05. Enriched GO terms were plotted using custom R scripts. Statistically significant DEGs were further analyzed for enrichment in biological processes (BP), cellular components (CC), molecular functions (MF), and Kyoto Encyclopedia of Genes and Genomes (KEGG) pathways. The top 20 enriched GO terms and KEGG pathways for upregulated/downregulated DEGs were summarized. Functional annotation of the entire gene set was also explored via Gene Set Enrichment Analysis (GSEA) using the GSEA Java application.

### 2.16 Piezo1 knockdown

For Piezo1 knockdown experiments, MIN6 cells were transfected with Piezo1-specific small interfering RNA (siPiezo1) or a corresponding non-targeting negative-control siRNA (siNC) using Lipofectamine 3000 (Invitrogen) according to the manufacturer’s instructions. The sequences of the siRNAs were as follows: siPiezo1 sense, 5′- CUC​AAG​UAC​UUC​AUC​AAC​U-3′; siPiezo1 antisense, 5′- AGU​UGA​UGA​AGU​ACU​UGA​G-3′. After transfection, cells were cultured under the indicated matrix-stiffness conditions.

### 2.17 Statistical analysis

Statistical analyses were performed using GraphPad Prism 10. Data are presented as mean ± standard error of the mean (SEM), unless otherwise indicated. The exact sample size (n) and number of independent experiments are provided in the corresponding figure legends. For comparisons between two groups, an appropriate two-tailed Student’s t-test was used when the assumptions of the test were satisfied. For comparisons involving more than two groups, one-way ANOVA followed by an appropriate *post hoc* multiple-comparison test was used. For longitudinal blood glucose measurements, two-way ANOVA with an appropriate multiple-comparison procedure was used where applicable. Statistical significance was defined as p < 0.05. Exact p values are reported where appropriate.

## 3 Results

### 3.1 Preparation and evaluation of 3D hydrogels with bionic matrix stiffness gradient

Micro-morphological analysis of the hydrogel cross-section using scanning electron microscopy (SEM) revealed that HAMA hydrogels, under varying light exposure times, exhibited a homogeneous porous network structure (Figure 1A). This structure enhanced the oxygen exchange efficiency and nutrient metabolism of encapsulated cells. As the UV crosslinking time increased from 10 s to 60 s, the hydrogel pore size decreased, while the pore density per unit area significantly increased, leading to a reduction in porosity (Figures 1B,C). At a crosslinking time of 60 s, the average pore size of the HAMA hydrogel was reduced to less than 100 μm, which inhibits cell infiltration and angiogenesis. Thus, crosslinking times between 10 s and 45 s yielded more suitable pore sizes for cell encapsulation and delivery applications. The compressive modulus was assessed using an MTS universal testing machine, and the Young’s modulus was calculated from the stress-strain curve. As the crosslinking time increased from 10 s to 45 s, the Young’s modulus of the HAMA hydrogel gradually increased, indicating enhanced stiffness. However, when the irradiation time exceeded 45 s, the modulus decreased, suggesting that excessive crosslinking may result in increased brittleness and reduced deformation resistance (Figures 1D,E). Importantly, the matrix stiffness of hydrogels in all experimental groups was within the physiological range of normal pancreatic tissue, and a 3D culture system with a biomimetic stiffness gradient was successfully established.

**FIGURE 1:**
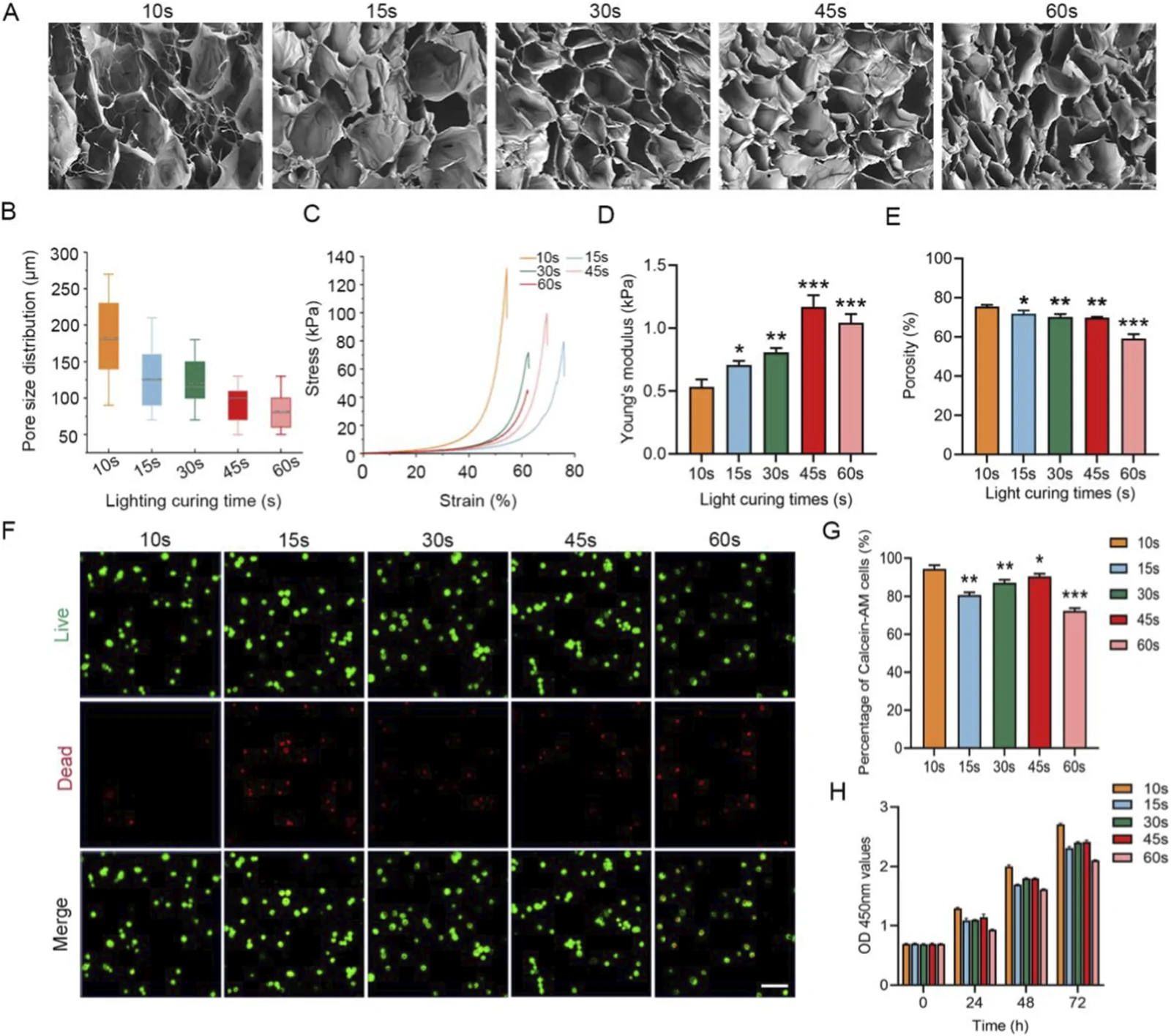
Characterization and biocompatibility evaluation of biomimetic matrix stiffness HAMA hydrogels. **(A)** Scanning electron microscopy images of hydrogel cross-sections from different groups. **(B)** Pore size distribution. **(C)** Stress-strain curves. **(D)** Young’s modulus. **(E)** Porosity (n = 6). **(F)** Live (green)/dead (red) cell assay to evaluate hydrogel biocompatibility (scale bar = 25 μm). **(G)** Proportion of live cells from panel F (n = 3). **(H)** CCK-8 assay showing the effect of HAMA hydrogel on MIN6 cell proliferation (n = 6). Data are presented as mean ± SEM. For quantitative analyses, n = 6 independent hydrogel samples for panels B–E, n = 3 independent biological experiments for panel G, and n = 6 independent wells for panel H. For comparisons among more than two groups, one-way ANOVA followed by Tukey’s multiple comparisons test was used. Statistical significance is indicated as *p < 0.05, **p < 0.01, and ***p < 0.001.

To assess the cytocompatibility of hardness gradient hydrogels, MIN6 cells were encapsulated in HAMA hydrogels with varying cross-linking times for *in vitro* culture. Cell viability was evaluated on the 5th day of culture using fluorescence staining of live and dead cells. Calcein acetoxymethyl ester (Calcein-AM), which freely permeates intact cell membranes, is hydrolyzed by esterases to produce green fluorescence, whereas PI selectively binds to the nucleus of necrotic cells with membrane rupture, emitting red fluorescence. Quantitative analysis of fluorescent labeling showed that over 85% of cells within the hydrogel were viable under cross-linking conditions of 10 s, 30 s, and 45 s, indicating good biocompatibility of the HAMA hydrogel (Figures 1F,G). Continuous cell proliferation monitoring through the CCK-8 assay (Figure 1H) demonstrated that, except for the inhibition of MIN6 cell proliferation in the 60 s hydrogel group, hydrogels in the other experimental groups showed a trend of cell proliferation. This finding was consistent with the live-dead staining results. Based on the mechanical properties and biocompatibility evaluations, the hydrogels prepared with 10 s and 45 s cross-linking times were selected for subsequent studies and designated as the soft matrix group and stiff matrix group, respectively.

### 3.2 Piezo1 is expressed on pancreatic islet cells

Given that bioelectric signaling is often coupled with the influx of calcium ions, it was hypothesized that membrane-localized expression of the mechanosensitive ion channel Piezo1 may be widespread in tissue cells involved in bioelectric activities linked to physiological functions. Immunofluorescence staining of mouse tissues, including heart, skin, skeletal muscle, and pancreas, confirmed that Piezo1 channel proteins exhibit a high-density distribution characteristic of pancreatic islet cell membranes (Figures 2A,C). Further bioinformatics analysis, using transcriptome data from the GEO database, showed that Piezo1 expression in different tissues of humans and mice is highly consistent (Figure 2B). Additionally, numerous mechanosensitive calcium channels are present in mammalian cell membranes. qPCR analysis of the relative expression of mechanosensitive ion channel mRNAs in MIN6 cells demonstrated that Piezo1 expression was significantly higher than that of other selected mechanosensitive channels, including Piezo2 and Trpv4, suggesting that Piezo1 may be an important candidate mechanosensitive ion channel involved in mechanical signal transduction in pancreatic β-cells (Figure 2D).

**FIGURE 2:**
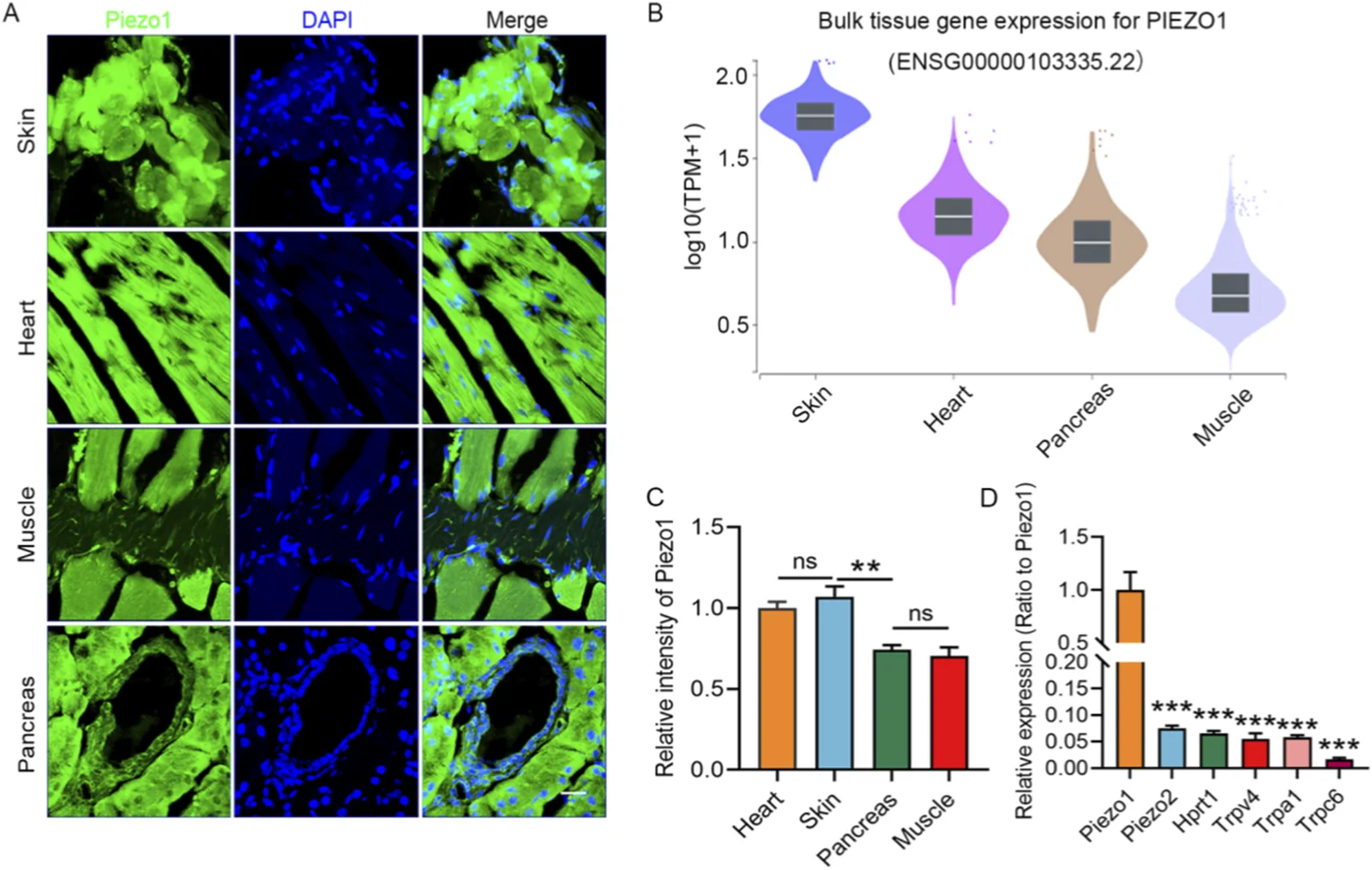
Piezo1 is widely distributed in pancreatic islets. **(A)** Immunofluorescence detection of Piezo1 expression in tissue samples (scale bar = 50 μm). **(B)** RNA-seq data for Piezo1 in skin, heart, pancreas, and skeletal muscle from human tissues. **(C)** Quantitative fluorescence analysis of panel A. **(D)** mRNA expression of mechanosensitive calcium channels on the cell membrane surface (n = 3). Data are presented as mean ± SEM from n = 3 independent biological experiments. For comparisons among more than two groups, one-way ANOVA followed by Tukey’s multiple comparisons test was used. Statistical significance is indicated as *p < 0.05, **p < 0.01, and ***p < 0.001.

### 3.3 Bionic matrix rigid microenvironment promotes Piezo1 expression and supports islet function

Islet tumor cells were encapsulated in soft and stiff matrix group hydrogels for culture, with traditional cell culture dishes (TCP) serving as the control. On the 5th day of culture, immunofluorescence and qPCR analyses revealed that Piezo1 expression in MIN6 cells cultured in the stiff matrix hydrogel group was significantly higher than in the control and soft matrix groups, with mRNA expression levels increasing 28-fold compared to the soft matrix group (Figures 3A–C). Notably, despite the Young’s modulus of TCP (∼1 GPa) being three orders of magnitude higher than that of the hydrogels, its induced Piezo1 expression was not statistically different from that in the soft matrix group. This may be attributed to (Zhang et al., 2021) the inability of the two-dimensional interface of TCP to effectively transmit biomechanical signals, and (Zanotelli et al., 2021) the mechanical overload caused by excessive matrix stiffness (>100 kPa), which may inhibit mechanosensitive channel function via a negative feedback mechanism. Using the Fluo-4 AM calcium fluorescent probe, it was observed that the average fluorescence intensity of intracellular Ca^2+^ in the stiff matrix group was 2.1-fold higher than in the soft matrix group (Figures 3D,E), suggesting that MIN6 cells respond to changes in hydrogel stiffness by activating Piezo1 channels, thereby mediating calcium influx.

**FIGURE 3:**
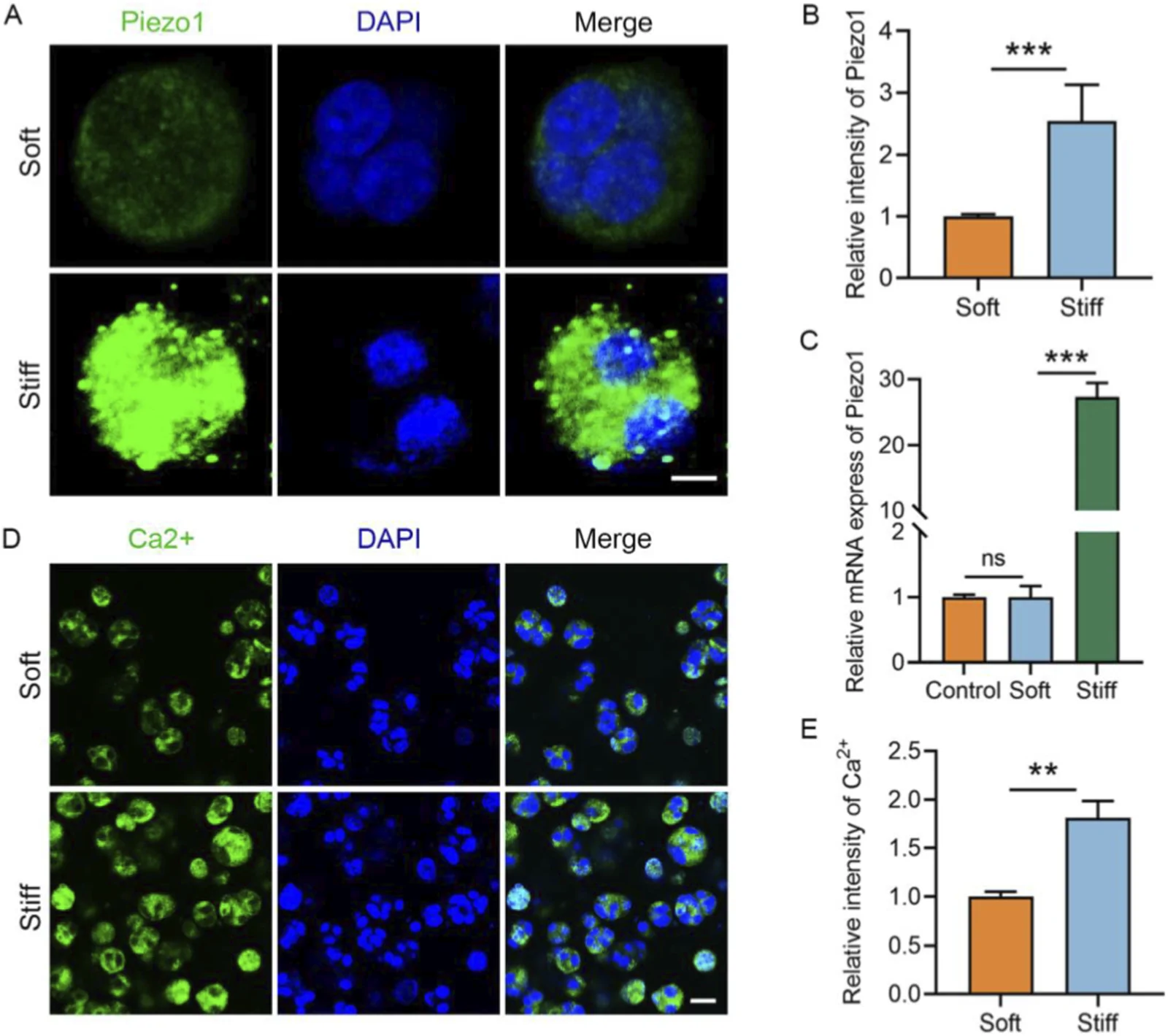
Matrix hardness promotes Piezo1 expression. **(A)** Cellular immunofluorescence detection of Piezo1 expression (scale bar = 25 μm). **(B)** Quantitative fluorescence statistics. **(C)** Piezo1 gene expression in different groups. **(D)** Calcium fluorescence probe detection of intracellular Ca^2+^ fluorescence intensity (scale bar = 100 μm). **(E)** Quantitative Ca^2+^ fluorescence statistics (n = 3). Data are presented as mean ± SEM from n = 3 independent biological experiments. For panels C, comparisons among the three experimental groups were performed using one-way ANOVA followed by Tukey’s multiple comparisons test. For panel B and E, the comparison between the soft- and stiff-matrix groups was performed using an unpaired two-tailed Student’s t-test. Statistical significance is indicated as *p < 0.05, **p < 0.01, and ***p < 0.001.

After culturing MIN6 cells in HAMA hydrogels for 5 days, quantitative immunofluorescence analysis revealed a significant increase in the fluorescence intensity of Insulin and Glucagon in the high matrix hardness group compared to the soft matrix group, with their average fluorescence intensity being approximately 1.5 times greater than that of the soft matrix group (Figures 4A–C). Real-time quantitative PCR analysis of key transcription factors involved in β-cell maturation (including Insulin, Pdx1, Mafa, and Nkx6-1) further confirmed that the stiff matrix hydrogel group significantly promoted the expression of these genes, suggesting that matrix stiffness enhances β-cell differentiation and function (Figures 4D–G). To evaluate the functional characteristics of the cells, GSIS experiments were conducted. Under both low and high glucose conditions, MIN6 cells cultured in the stiff matrix hydrogel group exhibited typical glucose concentration-dependent insulin secretion. Insulin secretion was elevated 1.4-fold and 1.7-fold compared to the soft matrix group under basal and high glucose stimulation conditions, respectively, indicating that the rigid matrix environment effectively enhances the glucose sensitivity and secretion capacity of β-cells (Figures 4H,I).

**FIGURE 4:**
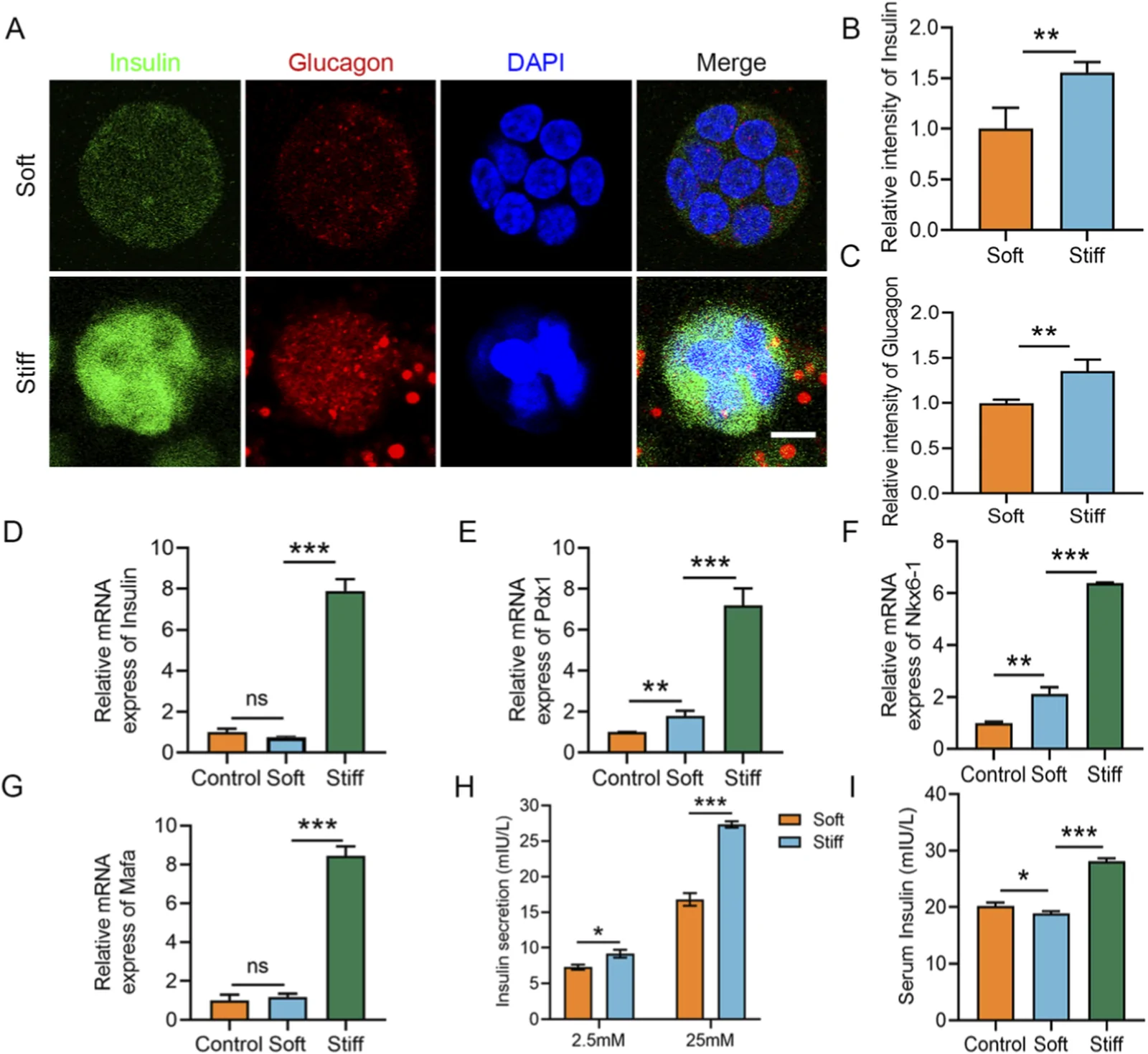
Effect of matrix hardness on islet function. **(A)** Cellular immunofluorescence detection of islet secretion function (scale bar = 25 μm). **(B)** Insulin fluorescence quantitative statistics. **(C)** Glucagon fluorescence quantitative statistics. **(D–G)** Expression levels of β-cell maturation-related transcription factors Ins (D), Pdx1 (E), Nkx6-1 (F), and Mafa (G). **(H)** Insulin concentration in cell culture supernatants. **(I)** GSIS assay for insulin secretion capacity of soft and hard hydrogels under low and high sugar conditions (n = 3). Data are presented as mean ± SEM from n = 3 independent biological experiments. For comparisons involving more than two groups, one-way ANOVA followed by Tukey’s multiple comparisons test was used. Statistical significance is indicated as *p < 0.05, **p < 0.01, and ***p < 0.001.

### 3.4 Piezo1 is associated with matrix stiffness-dependent islet function

To assess whether matrix stiffness affects islet function through Piezo1-mediated mechanical signaling, functional recovery experiments were conducted using pharmacological interventions. Treatment with the Piezo1 agonist Yoda1 (10 μM, 24 h) in the soft matrix group resulted in an increase in insulin immunofluorescence intensity from 72 ± 18 AU to 126 ± 24 AU, suggesting that Piezo1 activation partially mimicked the pro-insulin secretion effect of the stiff matrix (Figures 5A–C). In contrast, treatment with the mechanosensitive ion channel inhibitor GsMTx4 (2.5 μM, 24 h) in the stiff matrix group significantly decreased insulin fluorescence signals, even below the basal level observed in the soft matrix group. This suggests that, under physiological conditions, islet cells rely on the basal activity of Piezo1 channels to maintain functional homeostasis. qPCR analysis revealed that GsMTx4 treatment led to a significant reduction in the mRNA expression of Insulin, Pdx1, Mafa, and Nkx6-1 (Figures 5D–G), consistent with the immunofluorescence data, and demonstrated that suppression of gene expression adversely affected both insulin synthesis and secretion. These results suggest that matrix stiffness modulates islet function through the activity of the mechanosensitive channel Piezo1.

**FIGURE 5:**
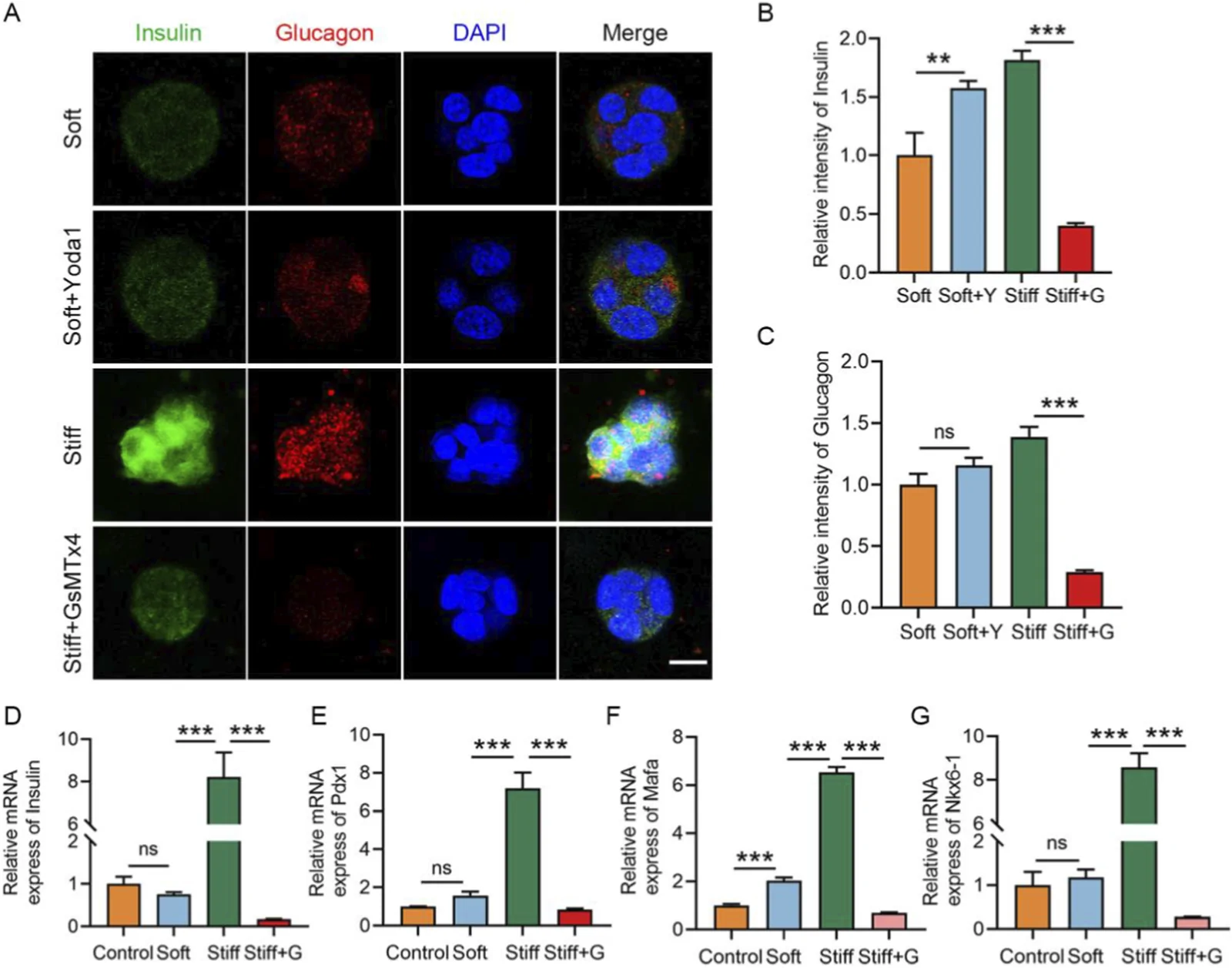
Matrix hardness regulates islet function through Piezo1 channels. **(A)** Cellular immunofluorescence detection of islet secretory function (scale bar = 25 μm). **(B)** Insulin fluorescence quantification. **(C)** Glucagon fluorescence quantification. **(D–G)** Expression levels of β-cell maturation-related transcription factors Ins (D), Pdx1 (E), Mafa (F), and Nkx6-1 (G) (n = 3). Data are presented as mean ± SEM from n = 3 independent biological experiments. Comparisons among experimental groups were performed using one-way ANOVA followed by Tukey’s multiple comparisons test. For comparisons between two groups, an unpaired two-tailed Student’s t-test was used where applicable. Fluorescence images were acquired under consistent imaging conditions within each experimental comparison, and predefined ROIs were used consistently for quantitative analysis. Statistical significance is indicated as *p < 0.05, **p < 0.01, and ***p < 0.001.

### 3.5 Analysis of potential mechanisms based on RNA-Seq and bioinformatics

To explore the molecular pathways associated with matrix stiffness, RNA-seq was performed on MIN6 cells encapsulated in soft and stiff HAMA hydrogels. Differentially expressed genes between the two groups were identified using DESeq2, followed by Gene Ontology (GO) and Kyoto Encyclopedia of Genes and Genomes (KEGG) enrichment analyses. The enriched pathways included HIF-related signaling and metabolic pathways associated with cellular energy metabolism (Figures 6A–E). These findings suggest that HIF-related signaling may be associated with the cellular response to matrix stiffness. However, because the present study did not directly manipulate HIF signaling or establish a molecular interaction between Piezo1 and HIF-related factors, these transcriptomic findings should be considered hypothesis-generating rather than evidence of a direct Piezo1-HIF regulatory pathway.

**FIGURE 6:**
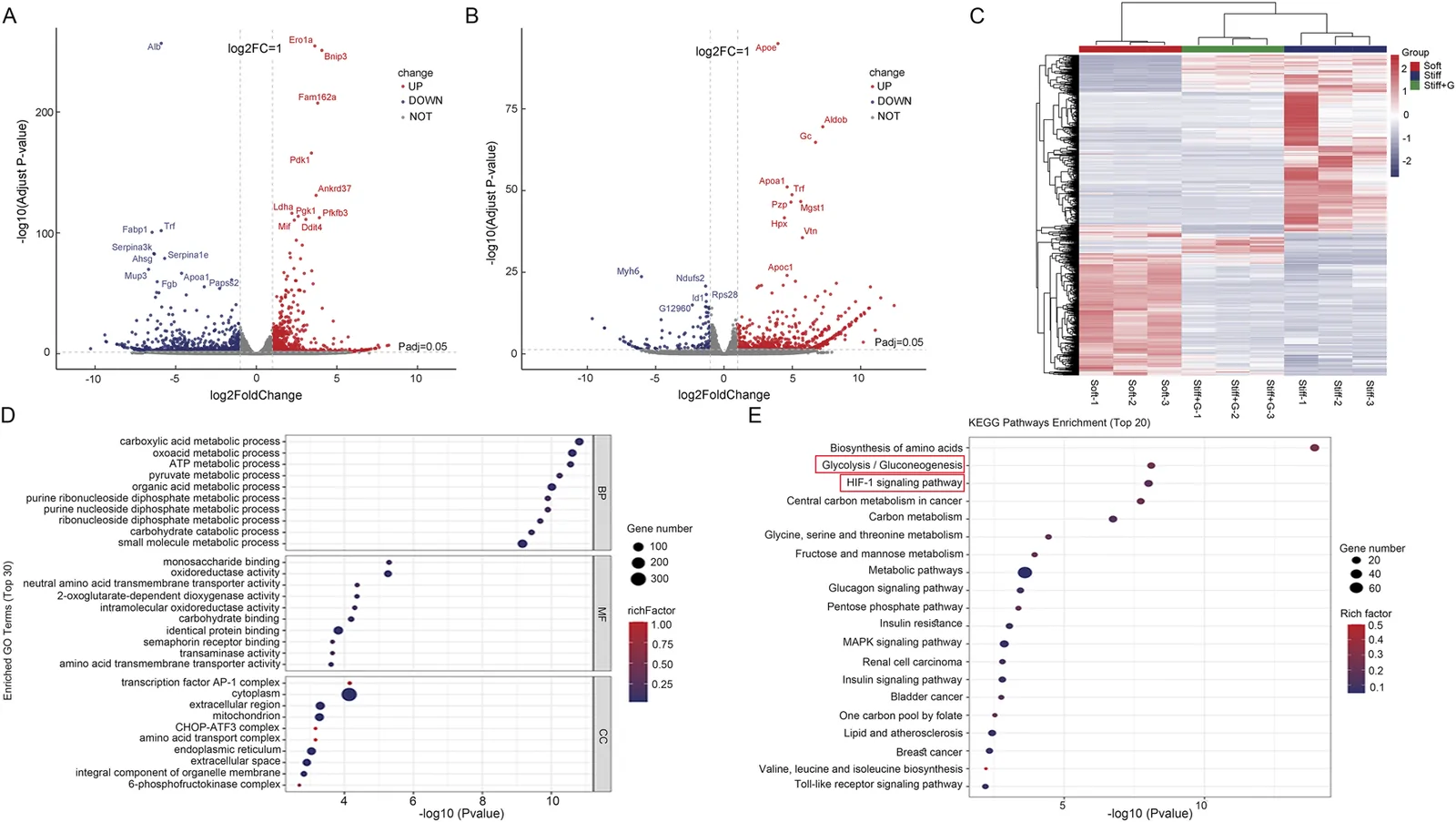
RNA-seq and molecular screening. **(A)** DEG volcano plot. **(B)** Top 10 molecules with the most significant marker differences. **(C)** Differential heatmap analysis. **(D)** GO enrichment. **(E)** Top 20 KEGG pathways.

### 3.6 Evaluation of *in vivo* transplantation results

At the end of transplantation, the hydrogels from the experimental group were removed, and the hearts, livers, spleens, lungs, and kidneys of the mice were harvested, ensuring compliance with ethical standards for small animal experiments. Histological examination of the organs revealed intact tissue structures, with no pathological necrosis or abnormal inflammatory infiltration. Additionally, paraffin sections of the organs were prepared, and H&E staining showed no evidence of nuclear consolidation, fragmentation, interstitial edema, fibrous hyperplasia, or significant inflammatory cell infiltration (Figure 7A), indicating that the HAMA hydrogel exhibited no cytotoxicity and demonstrated good *in vivo* biocompatibility. Furthermore, the removed hydrogels were observed, revealing a complete periosteum on the surface of the gels in all experimental groups, with neovascularization present around the gels (Figure 7B). Quantitative fluorescence analysis of the explanted grafts showed a significant increase in insulin fluorescence intensity in the stiff matrix hydrogel group, with the average fluorescence intensity reaching 3.2 times that of the soft matrix group. Insulin and glucagon levels were significantly lower in the stiff matrix group, even after Piezo1 knockdown, confirming that islet cell mechanosignaling primarily depends on Piezo1 activation (Figures 7C–E). The stiff matrix hydrogel group responded to *in vivo* hyperglycemia in diabetic mice earlier than the other two groups and was able to reduce blood glucose to within the normal range (Figure 7F). Throughout the transplantation period, both the soft and stiff matrix hydrogel groups effectively reduced blood glucose, maintained the mice’s body weight, and prolonged their survival time (Figures 7G,H).

**FIGURE 7:**
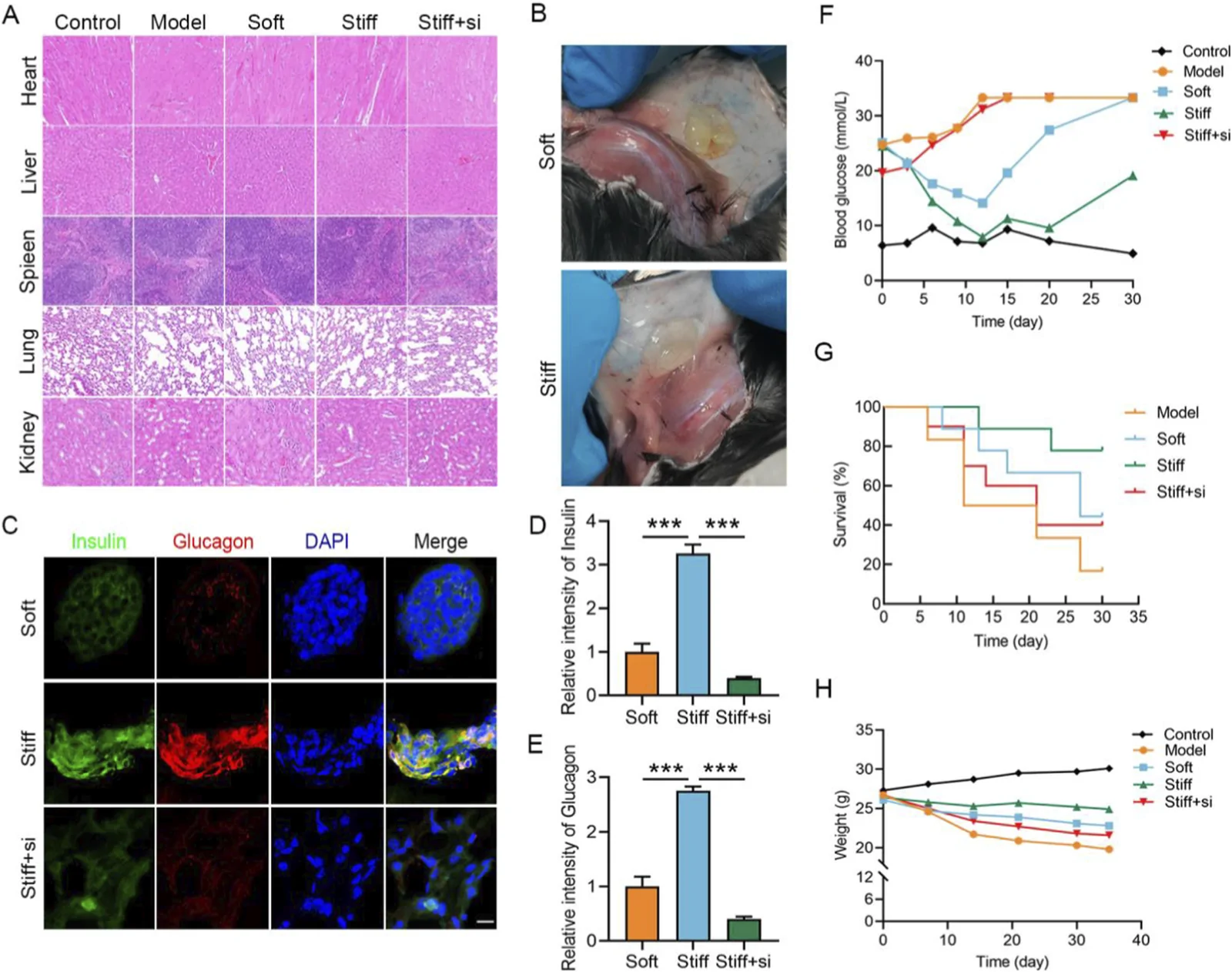
Evaluation of *in vivo* biocompatibility and transplantation efficacy. **(A)** Biosafety evaluation of the heart, liver, spleen, lung, and kidney in mice (scale bar = 100 μm). **(B)** representative macroscopic images of explanted HAMA hydrogels and surrounding tissue (n = 6). **(C)** Immunofluorescence detection of insulin and glucagon expression in explanted grafts (scale bar = 50 μm). **(D,E)** Quantitative analysis of insulin (D) and glucagon (E) fluorescence intensity. **(F)** Blood glucose levels after transplantation. **(G)** Survival curve. **(H)** Body weight changes (n = 6). Data are presented as mean ± SEM. n = 6 mice per experimental group. For comparisons among multiple groups, one-way ANOVA followed by Tukey’s multiple comparisons test was used where appropriate. Statistical significance is indicated as *p < 0.05, **p < 0.01, and ***p < 0.001.

## 4 Discussion

The ECM is a dynamic three-dimensional network composed of macromolecules such as collagen, elastin, and proteoglycans (Maneshi et al., 2021). It not only provides structural support for cells but also regulates biological processes such as cell proliferation, migration, differentiation, and apoptosis through mechanical signaling mediated by transmembrane receptors, including integrins and cadherins (Saraswathibhatla et al., 2023; Ge et al., 2021). Under physiological conditions, the ECM coordinates cellular behavior, maintains tissue homeostasis, and facilitates processes like damage repair by integrating biochemical and mechanical signals (Hadden et al., 2020; Mao et al., 2021). Increasing evidence suggests that the function of pancreatic β-cells is regulated not only by biochemical signals but also by the mechanical microenvironment within which the ECM is located. Thus, understanding the role of ECM mechanical signaling in regulating pancreatic islet function is critical (Llacua et al., 2018). To better understand the impact of ECM mechanical properties on the functional differentiation of pancreatic β-cells and insulin secretion, constructing an *in vitro* biomimetic model with mechanical responsiveness is essential.

Hydrogels offer distinct advantages in biomechanical signaling studies due to their biomimetic mechanical properties and dynamic responsiveness. As three-dimensional, network-structured polymer materials, hydrogels not only accurately replicate the viscoelastic characteristics of the natural ECM, including stress relaxation and creep behavior (Rehfeldt et al., 2007), but also enable precise regulation of mechanical properties through molecular design (Chaudhary et al., 2023; Chin et al., 2022). For instance, by adjusting the molecular weight and cross-linking density of alginate, the stress relaxation time can be reduced from 1 h to 1 min while maintaining a constant initial elastic modulus. This dynamic property directly influences the spreading, proliferation, and differentiation behavior of MSCs (Le et al., 2021). Compared to traditional rigid materials, hydrogels more closely match the mechanical properties of physiological tissues. Their fibrous network allows cells to remodel the matrix via integrin-mediated adhesion patches, activating mechanotransduction pathways such as FAK/RhoA/ROCK. This, in turn, regulates YAP/TAZ transcription factors in the nucleus at the molecular level (Qin et al., 2020; Liu et al., 2020). Moreover, the porous structure and adjustable stiffness of hydrogels provide an ideal platform for studying cellular mechanical responses in a three-dimensional microenvironment (Vahala et al., 2023; Patwardhan et al., 2021). Their biocompatibility and low immunogenicity make hydrogels suitable for maintaining long-term stable mechanical-biological interactions, which is essential when constructing organoid models or implantable biosensors (Heilala et al., 2023). These properties make hydrogels an invaluable tool for analyzing cellular mechanosensing mechanisms, developing smart biomaterials, and advancing regenerative medicine.

Piezo1 is a mechanosensitive, non-selective cation channel composed of three homologous subunits that form a propeller-like trimeric transmembrane protein (Saotome et al., 2018). Its unique structure allows it to respond directly to mechanical stimuli such as tensile force and fluid shear, converting mechanical signals into intracellular chemical or electrical signals by mediating the inward flow of cations like Ca^2+^, Na^+^, and K^+^. At the physiological level, Piezo1 is widely distributed across tissues and plays a regulatory role in multiple organs (Wu et al., 2017). It is highly expressed in vascular endothelial cells, renal epithelial cells, and cardiomyocytes, where it contributes to blood pressure regulation, urine osmolarity regulation, and cardiac mechano-chemical signaling (Jiang et al., 2023). In pancreatic islet β-cells, Piezo1 may also play a key role in regulating insulin secretion. The classical insulin release pathway involves membrane depolarization and inward Ca^2+^ flow triggered by glucose metabolism. Recognizing changes in cellular tone, Piezo1 is activated, initiating sustained Ca^2+^ influx (Lin et al., 2019; Komatsu et al., 1997; Ye et al., 2022). This calcium signaling not only enhances the cytosolic efficiency of insulin vesicles in coordination with glucose stimulation but may also upregulate genes related to insulin synthesis through activation of calmodulin-dependent kinase II (CaMKII).

In this study, two HAMA hydrogel models—soft and hard—were used to demonstrate that the stiff matrix significantly elevated Ca^2+^ concentration and insulin secretion in β-cells by activating Piezo1 channels. In contrast, an earlier study found that despite elevated Piezo1 expression in type 2 diabetic patients compared to healthy individuals, insulin release was reduced due to nuclear mislocalization caused by prolonged exposure to high glucose (Zhang et al., 2024). This discrepancy suggests that the biological effects of Piezo1 are tissue-specific and mechanistically threshold-dependent. Under physiological conditions, moderate mechanical stimulation can enhance insulin secretion by activating Piezo1, promoting transmembrane Ca^2+^ flow, while prolonged high-hardness stimulation may induce calcium overload and cellular dysfunction. It is noteworthy that the MIN6 cell line used in this experiment, although widely employed for β-cell function studies, exhibited only 60%–70% of the insulin secretion capacity seen in primary cells. This may be due to insufficient expression of glucose transporter protein (GLUT2) and low mitochondrial metabolic activity in MIN6 cells. Therefore, further validation of Piezo1’s regulatory role in primary islet cells or organoid models is needed to enhance the clinical translational potential of this study.

RNA sequencing revealed that, although a stiff matrix upregulated HIF-1α expression in insulin-secreting cells, the glycolytic targets of HIF-1 (e.g., Pfkfb3 and LDHA) were paradoxically increased in soft matrices. However, this glycolytic boost was not accompanied by enhanced insulin secretion, pointing to a decoupling of mechano-metabolic signaling (Hallahan et al., 2022). Soft matrix-induced Piezo1 underactivation likely limits ATP-dependent Ca^2+^ influx, while persistent glycolysis may elevate lactate and ROS, impairing mitochondrial function and insulin exocytosis (Yao et al., 2023). HIF-1 may also suppress β-cell identity transcription factors under soft conditions, further compromising secretory function. Together, these data suggest that matrix stiffness is associated with changes in both Piezo1-related Ca^2+^ signaling and HIF-related metabolic gene expression. However, the present data do not establish whether HIF signaling is directly downstream of Piezo1 or independently regulated by matrix stiffness.

Overall, our findings support an association between matrix stiffness, Piezo1-related Ca^2+^ signaling, and β-cell secretory function. Transcriptomic enrichment further identified HIF-related signaling as a candidate pathway associated with the stiffness-dependent response. Whether these pathways function independently or interactively requires further experimental validation.

## 5 Conclusion

This study established HAMA-based three-dimensional matrices with different stiffnesses and demonstrated that, under the experimental conditions tested, a stiff matrix enhanced intracellular Ca^2+^ accumulation and insulin-associated functions in MIN6 cells, accompanied by increased Piezo1 expression. Pharmacological modulation of mechanosensitive signaling further supported a potential contribution of Piezo1-associated signaling to the stiffness-dependent response, although Piezo1-specific causality could not be established using the current pharmacological approach. Transcriptomic analysis identified differential regulation of hypoxia-related and metabolic pathways, providing potential directions for future mechanistic investigation rather than definitive evidence of a downstream pathway. In a diabetic mouse transplantation model, stiff HAMA matrices containing MIN6 cells were associated with improved blood glucose control during the observation period. Overall, these findings indicate that matrix stiffness can modulate MIN6 cell insulin secretion and glucose-responsive function under defined experimental conditions.

## Data Availability

The original contributions presented in the study are included in the article/Supplementary Material, further inquiries can be directed to the corresponding authors.
