# Supplementary material for: The stiffness of three-dimensional biomimetic matrices regulates insulin secretion via the Piezo1 mechanosensitive ion channel
Source: Front Chem. 2026 Oct 9;14:1981529. doi: 10.3389/fchem.2026.1981529 (PMC13690767; doi:10.3389/fchem.2026.1981529)
Supplement: Supplementary file 1 [file DataSheet1.pdf]

Supporting Information

The stiffness of three-dimensional biomimetic matrices regulates insulin secretion via the Piezo1 mechanosensitive ion channel

Authors: Xi Chen[1, 2, †], Zhen Wang[1, †], Jiahao Gu[1, †], Yan Xue[3], Yang Xu[1], Yan Huang[1, *], Dongzhi Wang[1, *xc']

This file includes:

Table Sl

Table S1. List of all primer sequences used for qRT-PCR (Mus musculus).

Gene	Primer sequence (5’-3’)	length
Ins	F: AAACCATCAGCAAGCAGGTCA	21
	R: GCTTCCACCAAGTGAGAACCA	21
Pdx1	F: GACAACCTGGGCTCCAAACT	20
	R: CTGGTCCGTATTGGAACGCT	20
Mafa	F: AGGAGGAGGTCATCCGACTG	20
	R: CTTCTCGCTCTCCAGAATGTG	21
Nkx6-1	F: ACAAACCTCTGGACCCGAAC	20
	R: CACCGCTCGATTTGTGCTTT	20
Piezo1	F: CCTCTTCCTTTTCCAGGGGTTC	22
	R: GCCTCCCATACCATACTTGACAAT	24
Hprt1	F: CTGGTGAAAAGGACCTCTCGAAG	23
	R: CCAGTTTCACTAATGACACAAACG	24
Piezo2	F: GTAATCTGGTGGTGGCCCTC	20
	R: CACGCGACCATTGACTTTGG	20
Trpv4	F: ACAACACCCGAGAGAACACC	20
	R: TGAACTTGCGAGACAGATGC	20
Trpa1	F: GGAGCAGACATCAACAGCAC	20
	R: GCAGGGGCGACTTCTTATC	19
Trpc6	F: CGCTGCCACCGTATGG	16
	R: CCGCCGGTGAGTCAGT	16
β-Actin	F: GTGACGTTGACATCCGTAAAGA	22
	R: GTAACAGTCCGCCTAGAAGCAC	22
